# Retrospective review of pelvic malignancies undergoing total pelvic exenteration

**DOI:** 10.1186/1477-7819-10-110

**Published:** 2012-06-15

**Authors:** Maureen P Kuhrt, Ravi J Chokshi, David Arrese, Edward W Martin Jr

**Affiliations:** 1 Division of Surgical Oncology, Department of Surgery, Arthur G. James Cancer Hospital and Richard J. Solove Research Institute and Comprehensive Cancer Center, Wexner Medical Center, The Ohio State University, 395 W 12th Ave, Room 654, Columbus, OH, 43210, USA; 2 Division of Surgical Oncology, NJMS-UH Cancer Center, Newark, NJ, 07103, USA; 3 Columbus Surgical Specialists, Riverside Methodist Hospital, Columbus, OH, 43214, USA

**Keywords:** Total pelvic exenteration, Colorectal cancer, Locally advanced pelvic malignancy, Recurrent pelvic malignancy, R0 resection

## Abstract

**Background:**

In patients with locally advanced or recurrent pelvic malignancies, total pelvic exenteration (TPE) may be necessary for curative treatment. Despite improvements in mortality rates since TPE was first described, morbidity rates remain high due to the extensive resection and the aggressiveness of these tumors. We have studied the outcomes of TPE surgery performed at our institution.

**Methods:**

Fifty-three patients with various pelvic pathologies underwent TPE between 2004 and 2010. Patients were divided into two groups based on pathology: colorectal (*n =* 36) versus non-colorectal (*n =* 17) malignancies. Demographics, operative reports, pathology reports, periprocedural events, and outcomes were analyzed. Comparison of the two groups was performed using student’s *t*-test and Fisher’s exact test. Survival curves were constructed using the Kaplan–Meier method and compared using the log rank test.

**Results:**

The colorectal and non-colorectal groups were similar in demographics, operative times, length of stay, estimated blood loss, and rates of preoperative and intraoperative radiation use. Chemotherapy use was increased in the colorectal group compared with the non-colorectal group (55.6% vs. 23.5%, *P =* 0.04). Complication rates were similar: 86% in the colorectal group and 76% in the non-colorectal group. In the colorectal group, 27.8% of patients developed perineal abscesses, whereas no patients developed these complications in the non-colorectal group (*P =* 0.02). No survival difference was seen in primary versus recurrent colorectal tumors; however, within the colorectal group there was a survival advantage when comparing R0 resection to R1 and R2 resection combined. Median survival rates were 27.3 months for R0 resection and 10.7 months for R1 and R2 resection combined. The median survival was 21.4 months for the colorectal group and 6.9 months for the non-colorectal group (*P =* 0.002).

**Conclusions:**

Patients undergoing TPE for colorectal tumors have improved survival when compared with patients undergoing exenteration for pelvic malignancies of other origins. Within the colorectal group, the extent of resection demonstrated a significant survival benefit of an R0 resection compared with R1 and R2 resections. Despite TPE carrying a high morbidity rate, mortality rates have improved and careful patient selection can optimize outcomes.

## Background

In patients with advanced primary or recurrent pelvic malignancies without metastatic disease, extensive aggressive surgery such as total pelvic exenteration (TPE) may be necessary for curative treatment. TPE can also be considered in the setting of suspected disease recurrence, complications due to radiation treatment, and for palliative treatment of metastatic disease with severe pelvic symptomatology. Although initially performed at the Ellis Fischel Cancer Center in the 1940s, this highly morbid procedure was originally described by Brunschwig for patients with advanced pelvic malignancies in 1948 [1]. It involves a complete extirpation of the anal canal, rectum and distal colon, the bladder and lower ureters, the internal reproductive organs, draining lymph nodes, and the adjacent pelvic peritoneum. After removal of the bladder, methods of urinary diversion, such as the ileal conduit described by Bricker, [2] the double-barreled wet colostomy, [3] or neobladder construction are utilized. TPE has been performed in patients who have had primary and recurrent malignancies in the cervix, endometrium, vulva, vagina, prostate, bladder, rectum, and who have had pelvic sarcomas.

Early TPE series reported mortality rates of up to 30% [4-11]. More recent series have demonstrated improved mortality rates between 0 and 10% [12-17]. Despite a decrease in mortality, morbidity has remained high among these patients, with complication rates between 13 and 78% in patients undergoing TPE for colorectal malignancies [4,10-16,18-23]. Careful patient selection is imperative to optimize outcomes in patients undergoing this extensive surgical procedure for advanced and recurrent pelvic malignancies.

Our study describes the results of a single institution experience with TPE over a 6-year period, with a detailed examination of operative related complications. In addition, morbidity, mortality, and survival were evaluated for patients undergoing TPE for colorectal and non-colorectal malignancies and their results compared.

## Methods

Fifty-three patients underwent TPE between January 2004 and August 2010 at the Arthur G. James Cancer Hospital by surgeons from the divisions of surgical oncology, urology, and gynecologic oncology. This procedure was indicated for patients with locally advanced primary, recurrent, or suspected recurrent pelvic malignancies. After obtaining permission from The Ohio State University Institutional Review Board, the medical records of these patients were retrospectively reviewed.

TPE included resection of the distal sigmoid colon, rectum, anal canal, distal ureters, bladder, internal reproductive organs, and pelvic lymph nodes. Urinary diversion was achieved by construction of an ileal conduit [2] or a double-barreled wet colostomy [3,24]. In patients without bilateral abdominal wall violation and with patent inferior epigastric arteries, rectus abdominus myocutaneous flaps were used for pelvic reconstruction and closure. Preoperative imaging including computed tomography (CT) scans and/or positron emission tomography (PET) scans were used to assess resectability of the tumor and to determine if the malignancy was confined to the pelvis. Clinical signs of ureteral or bowel obstruction were not contraindications for TPE. TPE was not performed, however, if patients were too ill to tolerate an extensive surgery, disease was technically unresectable due to invasion of neurovascular structures, or if patients had widely metastatic asymptomatic disease. Palliative TPE was performed for patients with locally advanced unresectable disease or metastatic disease combined with significant pelvic symptomatology, including severe pelvic or perineal pain, obstruction, fistulas, bleeding, or non-healing perineal wounds causing significant morbidity and affecting quality of life. Patients were made thoroughly aware of the potential morbidities associated with the procedure and were willing to accept these risks in exchange for potential improvement in their pelvic symptoms.

TPE patients were divided into two groups, those who underwent TPE for colorectal cancer and those who underwent the surgery for non-colorectal cancer. Demographics including age, gender, and comorbidities were compared between the two groups. Preoperative treatment data including neoadjuvant chemotherapy and radiation were analyzed. Operative reports were reviewed to assess operative time, estimated blood loss, and administration of intraoperative radiation therapy. Intraoperative radiation therapy was indicated when there was concern of pelvic sidewall or sacral invasion by the tumor after gross tumor resection. It was contraindicated in patients who had already received a prohibitive dose of preoperative radiation to the pelvis, as determined by the consultant radiation oncologist. Periprocedural events, outcomes, and survival data were investigated for each of the two groups.

Tumors were categorized as primary, recurrent, or suspected recurrence. Suspected recurrences consisted of patients who had radiographic evidence of tumor recurrence with no pathological evidence of viable tumor on examination of the specimen. Operative and pathology reports of all patients were analyzed to determine the completeness of tumor resection. Completeness of resection was divided into R0 (complete resection of tumor), R1 (microscopic tumor in specimen margin), R2 (macroscopically incomplete resection), and suspected recurrence/complication. Postoperative and follow-up data collected included length of stay, morbidity, and mortality. Survival was calculated as the time from TPE to the time of death.

Descriptive statistics were used to summarize data. Continuous variables were compared by student’s *t*-test for comparison of independent groups and a non-parametric alternative (Wilcoxon rank sum test) for data not distributed normally. Contingency table analysis (chi-square and Fisher’s exact test) was used, when appropriate, for discrete data. A *P* value <0.05 was considered statistically significant. Statistical analysis was performed with SPSS Statistics 17.0 (SPSS Inc., Illinois, USA). Survival was calculated using the Kaplan–Meier method and compared using the log-rank test. Survival analysis was performed with JMP 9 (SAS Institute Inc., North Carolina, USA).

## Results

During the study period, 53 patients – 21 males and 32 females – underwent TPE for advanced primary, recurrent, or suspected recurrent pelvic malignancies. Based on pathology, patients were divided into a colorectal (*n =* 36) and a non-colorectal (*n =* 17) group for analysis. Demographics including age, gender, and number of comorbidities were similar between the two groups (Table 1). The median age of patients was 59 years (range 40–80) in the colorectal group and 52 years (range 38–79) in the non-colorectal group (*P =* 0.48). There were sixteen males and twenty females in the colorectal group, and five males and twelve females in the non-colorectal group (*P =* 0.37). Estimated blood loss, operative time, and length of stay were similar between the two groups. Mean length of stay was 17 days overall, 15.8 (± 9.9) days in the colorectal group and 19.5 (± 11.5) days in the non-colorectal group (*P =* 0.23). There was no significant difference in preoperative radiation and intraoperative radiation treatment rates between the two groups. Twenty-eight patients (78%) in the colorectal group and nine patients (53%) in the non-colorectal group underwent preoperative radiation treatment (*P =* 0.23). A majority of patients were referred to our institution for surgical intervention but received preoperative radiation at outside institutions. Preoperative radiation doses were available for 23 of the 37 patients. In the colorectal group, the 17patients for whom data was available received a mean dose of 5265 cGy preoperatively. In the non-colorectal group, data was available for six of the nine patients. They received a mean preoperative dose of 6990 cGy. Sixteen patients (44%) in the colorectal group and four patients (24%) in the non-colorectal group underwent intraoperative radiation treatment (*P =* 0.11). Significantly more patients (*n =* 20, 56%) underwent chemotherapy in the colorectal group compared with the non-colorectal group (*n =* 4, 24%) (*P =* 0.04).

**Table 1:** Demographics of patients with colorectal and non-colorectal tumors

**Characteristics**	**Colorectal** ***n =*** **36**	**Non-colorectal** ***n =*** **17**	***P*value**
Median age in years (range)	59 (40–80)	52 (38–79)	0.48
Gender M:F	16:20	5:12	0.37
Comorbidities	29	14	1.00
Mean (±SD) length of stay (days)	15.8 ± 9.9	19.5 ± 11.5	0.23
Mean (±SD) operative time (minutes)	603 ± 214	567 ± 165	0.53
Mean (±SD) estimated blood loss (liters)	1.9 ± 1.2	2.6 ± 2.2	0.16
Preoperative radiation	28 (78%)	9 (53%)	0.11
Intraoperative radiation	16 (44%)	4 (24%)	0.23
Chemotherapy	20 (56%)	4 (24%)	0.04

Overall, twelve patients (22.6%) had primary disease, thirty-four patients (64.2%) had recurrent disease, and seven patients (13.2%) had suspected recurrence or complications from previous therapy. Within the colorectal group, nine, twenty-two, and five patients had primary disease, recurrent disease, and suspected recurrence, respectively. Within the non-colorectal group, three, twelve, and two patients had primary disease, recurrent disease, and suspected recurrence, respectively. Table 2 shows the tumor pathology from all patients’ resections. TPE was indicated for thirty-six colorectal, six gynecologic, and five urologic malignancies. Three patients had leiomyosarcomas, two had anal cancer, and one patient had benign disease requiring TPE for extensive infection and inflammation.

**Table 2:** Tumor histology

Colorectal	36 (67.9%)
Gynecologic	6 (11.3%)
Urologic	5 (9.4%)
Leimyosarcoma	3 (5.7%)
Anal	2 (3.8%)
Benign	1 (1.9%)

Among both groups, there were nineteen R0 resections, twenty R1 resections, seven R2 resections, and seven resections of suspected recurrence/complication with no evidence of malignancy. Within the non-colorectal group, ten patients (58.8%) underwent R1 resection, which was significantly higher than in the colorectal group in which ten patients (27.8%) underwent R1 resection (*P =* 0.04) (Table 3). There was no significant difference between the two groups with regards to R0 and R2 resections, and suspected recurrence. Table 4 compares complications between the two groups. There was no difference in overall complications. The colorectal group had statistically significantly more perineal abscesses (*n =* 10, 28%) compared with the non-colorectal group (*n =* 0) (*P =* 0.02). Among the 53 patients, 17 underwent pelvic reconstruction with a rectus abdominus myocutaneous flap, whereas 36 underwent primary closure. There was no difference in operative time, blood loss, length of stay or overall wound complications between the two groups. In both groups, 47% developed pelvic abscesses. In those patients who underwent flap placement, two (12%) developed perineal abscesses, compared with eight (22%) in those who underwent primary closure (*P =* 0.47). Within the colorectal group, 13 patients underwent reconstruction with a rectus abdominus myocutaneous flap. Among the patients who underwent exenteration for colorectal cancer, two of thirteen (15.4%) with flap reconstruction developed a perineal abscess, compared with eight of twenty-three patients (34.8%) who underwent primary closure (*P =* 0.27).

**Table 3:** Extent of resection

**Resection status**	**Colorectal*n =*** **36**	**Non-colorectal*n =* 17**	***P *value**
R0	15 (41.7%)	4 (23.5%)	0.23
R1	10 (27.8%)	10 (58.8%)	0.04
R2	6 (16.6%)	1 (5.9%)	0.41
Suspected recurrence/ complication	5 (13.9%)	2 (11.8%)	1.00

**Table 4:** Morbidity and mortality following total pelvic exenteration for colorectal and non-colorectal tumors

**Complications (percent)**	**Colorectal*n =*** **36**	**Non-colorectal*n =* 17**	***P *value**
Overall	31 (86%)	13 (76%)	0.44
Wound infection	18 (50%)	6 (35%)	0.38
Wound dehiscence	8 (22%)	3 (18%)	1.00
Pelvic abscess	20 (56%)	5 (29%)	0.09
Perineal abscess	10 (28%)	0 (0%)	0.02
Enteric fistula	11 (31%)	3 (18%)	0.51
Sepsis	16 (44%)	5 (29%)	0.37
Bleeding	7 (19%)	1 (6%)	0.41
Respiratory failure	10 (28%)	2 (12%)	0.30
Deep venous thrombosis	7 (19%)	1 (6%)	0.41
30-day morbidity	29 (81%)	11 (65%)	0.31
30-day mortality	0 (0%)	0 (0%)	1.00

**Figure 1:**
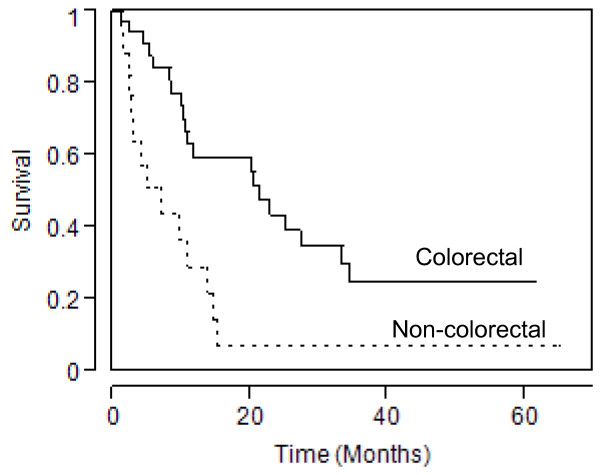
** Kaplan–Meier curve for survival in patients who underwent TPE for colorectal cancer compared with patients who underwent TPE for non-colorectal cancer (*P =*** **0.002).**

**Figure 2:**
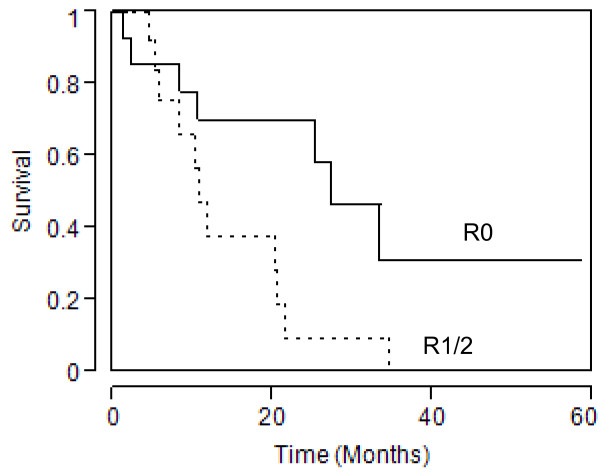
Kaplan–Meier curve for survival in patients who underwent TPE for colorectal cancer, based on extent of resection.

There was no 30-day mortality in either group. The median survival was 21.4 months in the colorectal group and 6.9 months in the non-colorectal group (*P =* 0.002) (Figure 1). Within the colorectal group, median survival was 27.3 months for the R0 group, compared with 10.7 months for the R1 and R2 groups combined (*P =* 0.03) (Figure 2). There was no significant difference in median survival between patients undergoing TPE for advanced primary colorectal cancer (25.2 months) and patients undergoing TPE for recurrent colorectal cancer (20.2 months).

## Discussion

Brunschwig initially described the technique of TPE as a palliative option for advanced cervical cancer [1]. The procedure has also evolved to become a treatment option for locally advanced and recurrent pelvic malignancies, including those of rectal, gynecologic, urologic, and sarcomatous origin. Since the initial description, mortality rates have improved from greater than 30% [4-11] to more acceptable rates of 0 to 10% [12-17]. Our series is consistent with other contemporary series, reflected in the 30-day mortality rate of 0% for tumors of both colorectal and non-colorectal origin. The decreased mortality rates over the past 60 years may be due to a variety of factors, including improved surgical technique, surgeon experience, radiological imaging, neoadjuvant chemoradiation therapy, anesthesia care, and perioperative care.

Our series demonstrated a statistically significant improved survival for patients undergoing TPE for cancer of colorectal origin compared with patients undergoing TPE for non-colorectal malignancies. Within the non-colorectal group, there were three patients with pelvic sarcomas. These tumors are rare, with prognosis based on the histologic grade and completeness of resection [25]. In our study, these patients lived for two, five, and fifteen months, respectively. Their poor survival is attributed to incomplete resections and high grade histologies. A review of the literature showed Ferenschild *et al.*’s group having a survival of 100% survival at 5 years [21]. Although they had a small number of patients, all four had undergone R0 resection. Histologies for the patients were not reported. For patients with sarcoma, chemotherapy does not provide any benefit, radiation is limited due to toxicity to adjacent structures, and surgery may be the only choice [25,26]. In a large cohort of patients with retroperitoneal sarcomas, Lewis *et al.* found that those who underwent incomplete resection (median survival of 18 months) had no difference in survival from those whose disease was unresectable, whereas those who underwent complete sarcoma resection had significantly improved outcome, with a median survival of 103 months [25]. Therefore, extensive surgical resection by means of a TPE may be a good option in patients with sarcoma, if they are able to undergo R0 resection.

In contrast to sarcomas, chemoradiation is the treatment of choice for locally advanced primary cervical malignancies. Most TPEs performed for gynecological cancer are performed for recurrent cervical cancer, [27-29] because up to one third of patients with primary cervical cancer have residual disease or develop recurrence after chemoradiation therapy [30]. For central recurrences or large central primary tumors not responsive to chemotherapy or radiation, radical surgery such as TPE may be the only potential cure. Five-year survival rates for patients who undergo TPE for recurrent cervical cancer have been similar to those for patients who undergo TPE for primary rectal cancer, with rates between 23% and 58% [21,27,28,31-36]. In our study population, six patients had gynecological malignancies: one vulvar, two vaginal, two cervical, and one endometrial. As part of the non-colorectal group, their median survival was poor, which is probably due to four of the six patients having an R1 resection status. Only two patients with gynecological malignancies underwent complete extirpation of tumor. This finding is consistent with the remainder of the non-colorectal group, which had significantly more patients with R1 resection status than the colorectal group. Although not statistically significant, there were fewer patients in the non-colorectal group (23.5%) than in the colorectal group (41.7%) that underwent complete tumor excision. This may explain the worse overall survival for the non-colorectal group. Within the heterogeneous non-colorectal group, the number of patients with each histology was too small for statistical analysis. This is consistent with much of the literature, which remains sparse regarding TPE for uncommon indications such as recurrent endometrial cancer and urological malignancies [21,34,36-38]. However, as with other recurrent pelvic malignancies, pelvic exenteration may be the only possible chance for cure. Barakat *et al.*’s study of 44 patients who underwent pelvic exenteration for recurrent endometrial cancer resulted in a 5-year survival rate of 20%, with a major morbidity rate of 80% [38]. Only 23 of these patients underwent total exenteration, whereas the others underwent less extensive resection via anterior or posterior exenteration. Future studies will require a larger number of patients with sarcomas, gynecological malignancies, and urological malignancies in order to make definitive conclusions regarding the use of TPE for treatment of these particular malignancies.

The importance of resection status is demonstrated both between the non-colorectal and colorectal groups, as well as within the colorectal group. In patients who underwent TPE for colorectal cancer, our results demonstrated a significant survival advantage for patients with R0 resection, with a median survival of 27.3 months, compared with those who had R1/R2 resection with a median survival of 10.7 months. This is consistent with previous studies that have demonstrated that complete resection is an important prognostic indicator of both improved disease-free and overall survival [13,17,20,21,39]. In patients who underwent pelvic exenteration for primary advanced or recurrent pelvic tumors, Zoucas *et al.* found that completeness of resection was the only variable with a significant impact on survival [17]. Kraybill *et al.*[40] described a 5-year survival rate of 44% in patients with negative margins, compared with a rate of 25% in patients with positive margins. Within our series, although curative resection was attempted, seven patients underwent R2 resection with evidence of gross tumor remaining in the pelvis. With large tumors in the pelvis requiring extensive dissection and mobilization, combined with neoadjuvant chemotherapy and/or radiation, it is sometimes difficult to determine whether complete resection may be achieved until it is attempted. However, each of these seven patients also had severe pelvic symptoms preoperatively that would have been possible indications for palliative intervention, including colovesical or enterovesical fistulas with chronic urinary tract infections, colovaginal fistulas, and chronic pelvic or perineal pain. In these circumstances, where complete resection is not technically possible but patients have severe pelvic symptomatology, palliative TPE, leaving gross disease behind, would be indicated. Patients must be well informed preoperatively about the risk of leaving gross disease behind, even when TPE with curative intent is performed.

Resection status may partially explain our results with regards to survival for primary and recurrent colorectal cancer. Our results did not demonstrate any difference in survival between patients undergoing TPE for primary advanced and recurrent colorectal tumors, with a median survival of 25.2 months and 20.2 months, respectively. Other series, however, have demonstrated improved survival for patients with cancers of primary colorectal origin compared with recurrent, with 5-year survival rates ranging from 30 to 77% for primary [4,5,10,13-16,21,22,37,39,41-44] and from 6 to 31% for recurrent disease [14,21,23,37,41,43-46]. Kecmanovic *et al.*[37] found median survival rates following TPE for primary and recurrent colorectal tumors were 50 months and 31 months, respectively. Their study does not report nodal status or resection status of the specimens. It is possible that more patients with primary colorectal tumors had node-negative disease and underwent complete R0 resection, compared with those patients with recurrent colorectal tumors, which could explain the improved survival in the group with primary disease. Law *et al.* described a 5-year survival rate of 64% in patients who underwent TPE for primary colorectal cancer, and a rate of less than 11% in those who underwent TPE for recurrent colorectal cancer. Only one of nine patients with recurrent malignancy lived longer than 24 months, and this patient was alive at the last follow-up at 47 months [13]. In their series, 27% of patients with primary rectal cancer had node-positive disease and 100% underwent R0 resection, whereas only 44% of patients in the recurrent rectal cancer group underwent R0 resection. The low percentage of patients with node-positive disease and the much improved R0 resection status in patients with primary disease compared with the patients with recurrent disease could explain the difference in survival between the two groups. Similarly, Ferenschild *et al.* reported 5-year survival rates of 66% and 8% for patients who underwent TPE for primary and recurrent rectal cancer, respectively [21]. R0 resection status was reported in a higher proportion of patients with primary disease (85%) than with recurrent disease (65%). The resection status may account for the improved survival in primary disease. By contrast, within our series, we did not find a significantly improved survival in patients undergoing TPE for primary disease compared with recurrent disease. A high proportion of patients with primary tumors (67%) had node-positive disease. In addition, an R0 resection was achieved in 67% and 41% of patients with primary and recurrent colorectal disease, respectively. The similarity in survival rates between those with primary and those with recurrent colorectal disease in our series may be due to this high percentage of patients with primary disease who were node-positive, and the similar percentages of patients who underwent R0 resection for primary and recurrent colorectal disease.

The group of seven patients who underwent TPE for suspected recurrence or complications from radiation therapy is an interesting subset of patients. Six of the seven had undergone preoperative radiation treatment to the pelvis. Five of these, as well as the one patient who did not undergo radiation, underwent chemotherapy prior to surgery. Of these patients, four had frozen section analysis that showed no evidence of malignancy. The other three patients had some type of fistula prior to surgery: enterocutaneous, colovaginal, or colovesical. In two patients, permanent specimen analysis revealed extensive necrosis with no viable tumor, consistent with treatment effect. The remaining pathological analyses showed densely fibrotic tissue or necrotic tissue. Although no viable tumor was found on pathological analysis, it is obvious that their previous malignancy and their preoperative treatment – chemotherapy, radiation, surgery, or a combination – contributed to the patients’ symptoms, with patients having fibrotic areas with associated fistulas, hydronephrosis, or other obstruction. Two of these patients died a year postoperatively, one died at two years, and the remainder are still alive two, four, six, and seven years postoperatively. In this group of patients, all had severe preoperative pelvic symptomatology; therefore, although their pathology was considered benign, or at least without viable tumor, their extensive symptomatology would have been the indication for TPE.

Morbidity rates were substantial among all patients, with similar rates of 86% and 76% in the colorectal and non-colorectal groups, respectively. These rates are high compared with those reported in the literature, which ranged from 13 to 78% [4,10-16,18-23]. However, our high rates can be explained by our stringent definition of morbidity, which included any deviation from the expected postoperative course, including anything that required longer hospitalization or any type of intervention. Rates of wound infection and wound dehiscence were similar between the groups. In the colorectal group, 50% of patients had wound infections compared with 35% in the non-colorectal group. Wound dehiscence occurred in 22% of colorectal patients and in 18% of non-colorectal patients. These accounted for the majority of the complications in both groups. There were significantly more perineal abscesses and a trend toward more pelvic abscesses in the colorectal group than in the non-colorectal group. The rate of pelvic abscesses in the non-colorectal group is consistent with gynecologic literature [27]. The comparatively higher abscess rate in the colorectal group is probably due to the location of the tumors and the extensive perineal and pelvic manipulation of the often irradiated, inflamed, or fixed bowel that is required to dissect and extirpate these tumors, with potential bacterial contamination from bowel contents. Within our study group of 53 patients, 17 underwent pelvic reconstruction with rectus abdominus myocutaneous flaps to assist with wound healing. Thirteen of these patients were within the colorectal group. There was no difference in complications between those who had flaps and those who underwent primary closure. There were fewer perineal abscesses in those with flaps, although this was not statistically significant. Among those who underwent TPE for colorectal malignancy, there were fewer patients who developed perineal abscesses with flap placement compared with those who had primary closure, but this also was not statistically significant. A larger study may demonstrate an advantage to using rectus abdominus myocutaneous flaps for closure of pelvic wounds following TPE for colorectal malignancy.

This study is limited by its retrospective nature, leading to the inability to establish a cause and effect relationship. It is also limited due to its small sample size. Within the non-colorectal cancer group, there were too few patients with each type of histology to make conclusions about patients who undergo TPE for any other particular type of pelvic malignancy, such as cervical cancer, endometrial cancer, or bladder cancer. A prospective study including a larger sample size of patients who have each of the various other pelvic cancers would allow us to make conclusions related to their respective malignancies. It would also be beneficial to include an assessment of quality of life, especially because our results showed such a short median survival for patients in the non-colorectal cancer group.

## Conclusions

Patients who undergo TPE for colorectal cancer have improved survival compared with patients who undergo this extensive surgery for pelvic malignancies of other origins. The results show improved survival for colorectal cancer patients with complete R0 resection of tumor. Although the procedure is still associated with significant morbidity, mortality rates are acceptable. Careful patient selection can optimize outcomes in patients undergoing TPE. Future studies may seek to predict which colorectal patients could potentially undergo TPE with successful R0 resection, leading to the best overall survival benefit from this procedure.

## Abbreviations

CT: Computed tomography; PET: Positron emission tomography; TPE: Total pelvic exenteration.

## Competing interests

The authors have no competing interests to declare.

## Authors’ contributions

MK participated in conception of the study, data collection and analysis, and drafted the manuscript. RC participated in conception of the study, data collection and analysis, and helped draft the manuscript. DA participated in conception of the study. EM participated in conception of the study, design and coordination, data analysis, and helped draft the manuscript. All authors read and approved the final manuscript.
